# Eczema of Ten Years’ Duration Cured with Mercurius-sol-h^5m^

**Published:** 1896-11

**Authors:** E. V. Ross

**Affiliations:** Rochester, N. Y.


### ECZEMA OF TEN YEARS' DURATION CURED WITH MERCURIUS-SOL-$H^{5m}$.

E. V. Ross, M. D., Rochester, N. Y.

In October, 1892, I was consulted by Mrs. A. K., æt. thirty-five; dark complexioned, short and thick set; had always been well except for a red, dry scaly eruption on the posterior surface of right thigh, which extended from the gluteal fold down to popliteal space and half way round on sides. She was much annoyed by the intense itching, which was worse at night when in bed. This eruption had existed for the past ten years, and during this period she had consulted a number of doctors, the last one was a very learned man connected with the Montreal General Hospital. He gave her ointment to apply that would "reduce the redness and allay the itching," but did not cure. In fact he said her disease was incurable and my patient had become quite reconciled to this belief. Her main object in consulting me was that she had "got out of ointment and thought that perhaps I could give some that would answer as well." I thereupon gave her Ointment? No! but a five minutes' discourse on palliation and cure, and incidentally touched upon Allopathy and ointments, at the conclusion of which I asked her if she was willing I should attempt to cure her. Said she: "If you think it possible." With this I took down a volume of Gentry's *Concordance Repertory*, and turned to the section on lower extremities, and then looked for eruption, and this is what I found: "Hepetic eruption on posterior surface of thigh. Merc." This with the nightly aggravation when in bed led me to administer Merc-sol-h.$^{5m}$ (fluxion), which I prepared on the Santee Gravity potentizer. I selected Merc-solubilis on account of its affinity for the skin. My patient was evidently much surprised when I dropped a few sugar pellets on her tongue and gave her some blank discs in a two-drachm phial, for she remarked: "Is that all?" "Yes, except my fee," which, strange to say, she paid, and with half doubting smile departed.

October 14th.—One week later she called and gave the following report: there was not any apparent change in the eruption, but the itching was decidedly less. ℞ Merc-sol-h.$^{5m}$ and placebo to follow.

October 22d.—Decided change. Upon inspection I found there was less redness and the skin presented a smoother appearance. ℞ Sac-lac.

October 30th.—Rash gone; there is but a slight redness remaining. Sac-lac.

November 9th.—Skin presents a natural appearance, except a spot of redness just above popliteal space. ℞ Sac-lac. This was her last prescription. She has remained free from any eruption to date, October 15th, 1896, and I think we can safely say cured. It may seem needless to add that this lady is now a firm believer in the virtues of a little sweet medicine and Homœopathy.

